# Modeling of the Renal Kinetics of the AT1 Receptor Specific PET Radioligand [^**11**^C]KR31173

**DOI:** 10.1155/2013/835859

**Published:** 2013-09-08

**Authors:** Nedim C. M. Gulaldi, Jinsong Xia, Tao Feng, Kelvin Hong, William B. Mathews, Dawn Ruben, Ihab R. Kamel, Benjamin M. W. Tsui, Zsolt Szabo

**Affiliations:** ^1^Russell H. Morgan Department of Radiology and Radiological Science, Johns Hopkins Medical Institutions, Baltimore, MD 21287, USA; ^2^Department of Molecular and Comparative Pathobiology, Johns Hopkins Medical Institutions, Baltimore, MD 21287, USA

## Abstract

*Purpose*. The radioligand [^11^C]KR31173 has been introduced for PET imaging of the angiotensin II subtype 1 receptor (AT1R). The purpose of the present project was to employ and validate a compartmental model for quantification of the kinetics of this radioligand in a porcine model of renal ischemia followed by reperfusion (IR). *Procedures*. Ten domestic pigs were included in the study: five controls and five experimental animals with IR of the left kidney. To achieve IR, acute ischemia was created with a balloon inserted into the left renal artery and inflated for 60 minutes. Reperfusion was achieved by deflation and removal of the balloon. Blood chemistries, urine specific gravity and PH values, and circulating hormones of the renin angiotensin system were measured and PET imaging was performed one week after IR. Cortical time-activity curves obtained from a 90 min [^11^C]KR31173 dynamic PET study were processed with a compartmental model that included two tissue compartments connected in parallel. Radioligand binding quantified by radioligand retention (80 min value to maximum value ratio) was compared to the binding parameters derived from the compartmental model. A binding ratio was calculated as DVR = DV_S_/DV_NS_, where DV_S_ and DV_NS_ represented the distribution volumes of specific binding and nonspecific binding. Receptor binding was also determined by autoradiography *in vitro*. *Results*. Correlations between rate constants and binding parameters derived by the convolution and deconvolution curve fittings were significant (*r* > 0.9). Also significant was the correlation between the retention parameter derived from the tissue activity curve (*Y*_ret_) and the retention parameter derived from the impulse response function (*f*_ret_). Furthermore, significant correlations were found between these two retention parameters and DVR. Measurements with PET showed no significant changes in the radioligand binding parameters caused by IR, and these *in vivo* findings were confirmed by autoradiography performed *in vitro*. *Conclusions*. Correlations between various binding parameters support the concept of the parallel connectivity compartmental model. If an arterial input function cannot be obtained, simple radioligand retention may be adequate for estimation of *in vivo* radioligand binding.

## 1. Introduction

**Figure 1:**
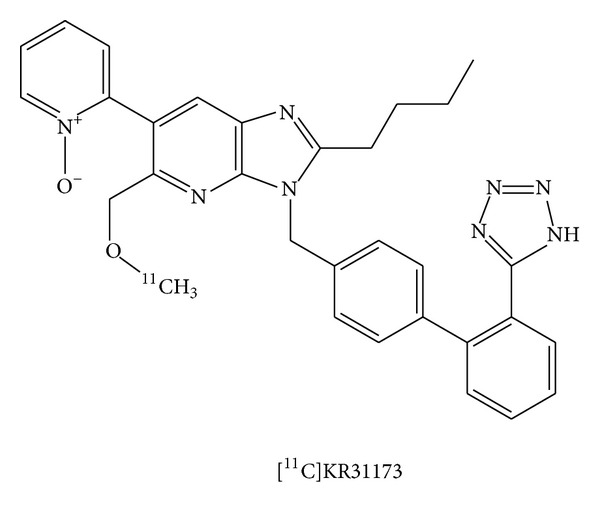
Chemical structure of [^11^C]KR31173.

Imaging the angiotensin II subtype 1 receptor (AT1R) with positron emission tomography (PET) has been achieved with the radioligand [^11^C]KR31173 (2-butyl-5-[^11^C] methoxymethyl-6-(1-oxopyridin-2-yl)-3-[[2′-(1H-tetrazol-5-yl)biphenyl-4-yl] methyl]-3H-imidazo[4,5-b] pyridine; Figure 1) [1]. Radioligand retention derived from the resulting impulse response function has been used for quantification of radioligand binding in the kidneys [2]. Until now, no compartmental model has been proposed. In the presented paper, we used a compartmental model with two tissue compartments connected in parallel for processing of PET studies. Goal was to validate the feasibility of retention parameters of radioligand binding *in vivo*.

The purpose of the present work was to calculate radioligand retention using both the tissue time-activity curve and the impulse response function and correlate these retention parameters with the compartmental radioligand kinetic parameters in an animal model of renal ischemia followed by reperfusion. Due to lack of a transfer barrier in the kidneys, a parallel connectivity model was applied for curve fitting and parameter estimation. This is different from tracer kinetic models typically applied for brain receptor imaging where the presence of the blood brain barrier makes a serial connectivity model more applicable [3, 4].

Acute renal ischemia followed by reperfusion (IR) is a clinical problem often associated with kidney transplantation, hypoperfusion during surgery, acute infarction, and/or trauma of the organ [5, 6]. The molecular mechanism of tissue injury includes angiotensin II (Ang II) and the angiotensin subtype 1 receptor (AT1R) [7]. Previously, our group has shown that AT1R binding was upregulated in renal ischemia [2] and in myocardial IR [8]. This is the first study of the effects of renal IR on AT1R binding *in vivo*.

## 2. Materials and Methods

### 2.1. Animal Model: Ischemia Followed by Reperfusion (IR)

The study protocol was approved by Johns Hopkins University Institutional Animal Care and Use Committee. Ten domestic pigs with an average age of 3 months and average weight of 23 kg were included in the study. Five sham animals (controls) received angiography without arterial occlusion. Five pigs were subjected to acute occlusion followed by reperfusion (IR). In both groups, vascular surgical cutdown under general anesthesia was performed to access the common femoral artery in the right groin of each animal. A pigtail catheter (5F or 6F; Cook, Inc.) was placed into the left renal artery. Basal contrast angiography was performed to evaluate the renal arteries and the status of the vascular supply to the kidneys. For the IR group, an endovascular dilatation catheter with a 3–5 mm diameter (Cordis, Johnson & Johnson Co., ref. 438-5020S) was used to occlude the left renal artery via balloon inflation.

The occlusion lasted sixty minutes to achieve acute ischemic conditions. After that time, the balloon was deflated to achieve reperfusion. Contrast angiography was performed before, during, and after balloon occlusion in the IR group or before and after catheter insertion in the sham group (*Infinix Celeve* IS Angiographic X-ray Systems; *Toshiba*, *Tokyo*, Japan). Heparin (2500 units) was injected before the balloon occlusion to prevent renal artery thrombosis from intimal injury. MRI and PET were performed one week after IR or after sham to give a chance to reveal changes in receptor level if any. Blood chemistries, urine specific gravity and PH values, angiotensin II, aldosterone, and plasma renin activity (PRA) were measured on the day of PET imaging.

### 2.2. MRA Imaging

Renal blood flow was evaluated by angiography, MRA, and O-15 water PET. Contrast angiography and MRA angiography images were analyzed by visual inspection. Perfusion changes were quantified by O-15 water PET. T1 weighted MRA images were obtained before and after gadodiamide injection with a 1.5 T CV/i MR scanner (GE Medical Systems, Signa HDe, Excite, USA) [2]. The animals were on mechanical ventilation, and the ventilator was stopped for the acquisition of each MR image to minimize the risk of respiratory artifacts. After gadodiamide, the MR scans were reconstructed and displayed as maximum intensity projection (MIP) images.

### 2.3. PET Imaging

After completion of the MRI scan, two dynamic PET scans were obtained using a GE Advance PET scanner, one with [^15^O]H_2_O and one with [^11^C]KR31173 as previously published [1]. Animal positioning for PET was determined based on the MRI scans. Before PET, a transmission scan was obtained with a pair of 370 MBq germanium-68 pin sources.

After fast intravenous injection of 740 MBq (20 mCi), [^15^O]H_2_O the image sequence consisted of twenty-four 5 sec frames followed by six 10 sec frames. [^11^C]KR31173 was administered IV at a dose of 551–762 MBq (15–21 mCi) and specific activity of 81 ± 33 GBq/*μ*mole (2200 ± 900 mCi/*μ*mole). The receptor PET study sequence included 57 scans in 90 minutes with the shortest time of 5 seconds at the beginning and the longest time of 5 minutes at the end. PET scans were reconstructed with filtered back projection at a voxel size of 2 × 2 × 4 mm. Image smoothing was applied only for display but not during quantitative analysis.

Time-activity curves (TACs) were generated using the MIPAV software (Medical Image Processing, Analysis and Visualization; Center for Information Technology, National Institutes of Health, Bethesda, MD) with delineation of the kidney contour at 20% of maximal pixel activity within the organ using color threshold method. TACs were corrected for isotope decay and injected dose and were expressed in units of Bq/mL/MBq (nCi/mL/mCi) in order to minimize effects of organ size.

The input function required for compartmental and for noncompartmental (deconvolution) analyses was obtained from metabolite corrected arterial plasma samples. For this purpose, blood samples of 0.5 mL at increasing steps from 5 sec to 5 min were collected from the carotid artery, centrifuged, counted in a well-type counter (Compugamma CS Universal Gamma Counter, LKB), and cross-calibrated with the image data from the PET scanner.

Additional 5 mL arterial samples were obtained at 0, 5, 15, 30, 60, and 90 minutes after injection to estimate unmetabolized plasma radioligand concentration by the column-switch high-performance liquid chromatography method [9].

### 2.4. Perfusion Ratios

Renal cortical TACs derived from the [^15^O]H_2_O PET scans were used to evaluate renal perfusion. To obtain the perfusion ratio, the area under the curve (AUC) of each TAC was obtained within the time window defined by the time of onset of activity and the time of first peak activity of the two kidneys. The perfusion ratio between the IR kidney and contralateral kidney was calculated based on the assumption that the contralateral kidney had normal perfusion and could serve as a reference. For the contralateral kidney a perfusion ratio of 1 was used. For control kidneys, the AUC ratio between individual kidneys and the average of bilateral kidneys were calculated.

### 2.5. Tracer Kinetic Model for [^11^C]KR31173

Tissue radioligand activity was described as a sum of two activities: one including arterial activity *C*_*A*_(*t*) weighted by the organ blood volume (BV) and another including proper tissue activity weighted by (1 − BV) and calculated as a convolution integral (⊗) of the arterial input function *C*_*A*_(*t*) with the tissue impulse response function *f*(*t*):

(1) $$\begin{array}{c} \text{PET}\left(t\right)=\text{BV}\times C_{B}\left(t\right)+\left(1-\text{BV}\right)C_{A}\left(t\right)⊗f\left(t\right). \end{array}$$

A predetermined value of 0.15 was used for BV. This value was obtained in a separate group of four pigs by fitting the renal cortical time-activity curves within a shorter time interval of 12 minutes with inclusion of BV as the fifth unknown compartmental model parameter.

**Figure 2:**
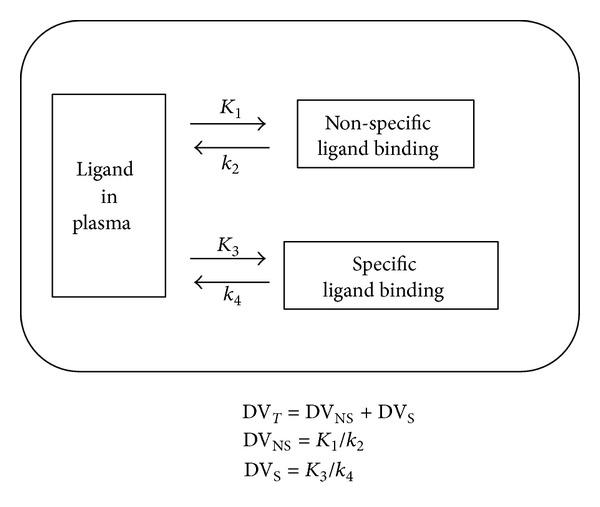
Compartmental model. The rate constants *K*_1_ and *k*_2_ represent binding to and release from nonspecific binding sites. The rate constants *K*_3_ and *k*_4_ represent binding to and release from specific binding sites. With the proposed parallel connectivity model, the distribution volume of binding is DV_*T*_ = DV_S_ + DV_NS_, where DV_*T*_ is total distribution volume, DV_S_ is distribution volume of specific binding, and DV_NS_ is distribution volume of nonspecific binding. Each DV is represented by the area under curve of the corresponding component of the impulse response function (*K*_1_/*k*_2_ versus *K*_3_/*k*_4_).

Radioligand concentrations were defined by a system of differential equations (2) that described nonspecific binding *C*_NS_(*t*) and receptor specific binding *C*_S_(*t*):

(2) dCNS(t)dt=K1CA(t)−k2CNS(t),dCS(t)dt=K3CA(t)−k4CS(t),

where *K*_1_ and *k*_2_ are compartmental rate constants of ligand exchange with nonspecific binding sites while *K*_3_ and *k*_4_ are compartmental rate constants of ligand exchange with specific binding sites (Figure 2). The compartmental model described in (2) is based on the following assumptions: (a) arterial blood and tissue are connected in series, (b) tissue compartments are connected in parallel, (c) radioligand binding affinity at specific binding sites is always higher than the binding affinity at nonspecific binding sites in the same kidney, and (d) there is no exchange between specific binding and nonspecific binding. Therefore, the renal parenchymal impulse response function is a simple biexponential:

(3) $$\begin{array}{c} f\left(t\right)=K_{1}e^{-k_{2}t}+K_{3}e^{-k_{4}t}. \end{array}$$

With the parallel model, the area under the curve of each exponential function represents each distribution volume. Thus, DV_S_ = *K*_3_/*k*_4_ represents the distribution volume of specific binding, and DV_NS_ = *K*_1_/*k*_2_ represents the distribution volume of nonspecific binding. Total distribution volume is the sum of two individual distribution volumes:

(4) $$\begin{array}{c} \text{D}{\text{V}}_{T}=\text{D}{\text{V}}_{\text{S}}+\text{D}{\text{V}}_{\text{NS}}. \end{array}$$

A specific-to-nonspecific binding ratio DVR was calculated as DV_S_/DV_NS_.

The Levenberg-Marquardt algorithm [10, 11] was implemented to estimate parameters *K*_1_, *k*_2_, *K*_3_, and *k*_4_, with two approaches: a deconvolution approach and a convolution approach. For the deconvolution approach the impulse response function was calculated by deconvolution, and subsequently the compartmental rate constants were estimated by fitting the impulse response function between 2 and 80 minutes after injection. For the convolution approach fitting was performed on measured time-activity curves in the same time interval. For both approaches (i.e., convolution and deconvolution) the impulse response function was used in its analytical form (3). Processes of deconvolution and convolution were carried out in frequency domain as follows. For convolution the response function (time-activity curve; *y*(*t*)) was described as the convolution integral (⊗) of the input function *x*(*t*) with the impulse response function *f*(*t*):

(5) $$\begin{array}{c} y\left(t\right)=x\left(t\right)⊗f\left(t\right). \end{array}$$

Equation (6) represents (5) in frequency domain:

(6) $$\begin{array}{c} \psi =\xi \phi . \end{array}$$

Frequency-domain deconvolution analysis [12] included regularization by the third difference operator function **c** = [1, −3,3, −1,0,…, 0]:

$$\begin{array}{cc} \left(6\right) & \phi =\frac{\xi^{*}\psi }{\xi^{*}\xi +\kappa \kappa^{*}}. \end{array}$$

For this equation *κ* was the Fourier transform of **c**, the symbol * represented complex conjugate of the Fourier transforms, and *ϕ*, *ξ*, and *ψ* were the Fourier transforms of *f*(*t*), *x*(*t*), and *y*(*t*) calculated by fast Fourier transformations (FFTs).

### 2.6. Hormone Levels and Blood Chemistries

On the day of PET imaging blood and urine samples were collected for determination of angiotensin II, plasma renin activity, aldosterone, blood cell counts (white blood cell count, red blood cell count, and hemoglobin), chemistries (alkaline phosphatase, serum glutamic pyruvic transaminase, serum glutamic oxaloacetic transaminase, serum creatine kinase, serum urea nitrogen, and serum creatinine), and urine specific gravity and PH values. The purpose of these determinations was to search for any alterations in global kidney or liver function that could have a potential effect on the renin angiotensin system and the kinetics of the radiopharmaceutical.

### 2.7. Tissue Analysis

Each animal (10 total: five controls and five IRs) was euthanized with pentobarbital at the end of the study. Both kidneys were extracted within 10 minutes of euthanasia and stored at −80°C for subsequent autoradiography. Each kidney was sectioned in a microcryostat at −20°C into 60–80 *μ*m slices that were then mounted on polylysine-coated slides (Polysine, Erie Scientific).

Autoradiography was carried out in an assay medium containing 150 mmol/L of NaCl, 1 mmol/L of EDTA, 0.1 mmol/L of bacitracin, and 50 mmol/L of NaPO_4_ (pH 7.2). Tissue sections were preincubated for 30 minutes in the assay medium at 22°C to 24°C. The tissue sections were then incubated for 2 hours with [^125^I]-[Sar^1^Ile^8^] angiotensin II (Ang II) in the presence or absence of the subtype selective angiotensin AT2R receptor antagonist PD-123,319 (1 *μ*mol/L) in order to determine total binding and specific binding as described previously [13]. The sections were then rinsed sequentially in water and assay medium without radioligand, rinsed again in water, and dried under a stream of cool air.

Autoradiography was performed on a Beta Imager (2000Z, Biospace, Paris, France). Digital autoradiography images were analyzed by using the software *β*-Vision v.2.0. Each measurement was performed in triplicate.

### 2.8. Hypotheses and Statistical Analysis

Differences in the parameters estimates for the three groups, ischemia reperfusion kidneys (IR), contralateral kidneys (CL), and control kidneys (C) were tested. *In vitro* parameters included total binding and AT1R specific binding. *In vivo* binding parameters included *K*_1_, *k*_2_, *K*_3_, *k*_4_, DV_NS_, DV_S_, and DVR and were obtained by the convolution and deconvolution approaches. Retention parameters were calculated from the measured tissue activity curve (*Y*_ret_) and from the impulse response function (*f*_ret_).

Group statistics were characterized as mean ± standard deviation. Two way comparisons were tested with simple and paired *t*-tests; three way comparisons were tested with analysis of variance (ANOVA).

The *first hypothesis* was that there would be a positive correlation between rate constants obtained with the convolution and deconvolution techniques and between the various *in vitro* and *in vivo* binding parameters. This hypothesis was tested by one tailed Pearson correlation.

The *second hypothesis* was that imaging times can be shortened with less than 10% bias in the binding parameters DVR and *Y*_ret_. The effect of imaging time on the distribution volume ratio DVR_*r*_ obtained with the convolution approach at each time point *t* was compared with the value obtained with the total imaging time (midscan time point 87.5 min, total imaging time 90 min) as a reference. For this test DVR was calculated for the following midscan time points: 42.5, 47.5, 52.5, 57.5, 62.5, 67.5, 72.5, 77.5, and 82.5 min, and for the reference time point of 87.5 min. The bias of DVR was obtained at each time point *t* as

(7) $$\begin{array}{c} {\text{BIAS}}_{\text{DVR},t}=\frac{1}{12}\sum_{m=1}^{12}\frac{\left(\text{DV}{\text{R}}_{t}-\text{DV}{\text{R}}_{87.5}\right)}{\text{DV}{\text{R}}_{87.5}}. \end{array}$$

BIAS_*Y*_ret__ was obtained for each of the time points in the same manner. The DVR values for the last time point from all 12 kidneys were considered to represent a true pattern while the DVR values from the other 9 time points were investigated by factor analysis whether they would display the same pattern. Factor analysis is a computational technique employed to uncover hidden structures (patterns) within a dataset based on mutual correlations of the data. The dataset in this work was represented by all measurements of *Y*_ret_ and DVR obtained at the 10 listed time points. Factor analysis was employed as a confirmatory test whether the binding parameter (*Y*_ret_ or DVR) could be reproduced at these 10 imaging time points. DVR and *Y*_ret_ values obtained for the 10 time points were analyzed separately by principal component based factor analysis by reducing the ten measurements to a linear combination of 2 factors and 2 factor load vectors.

The *third hypothesis* was that the relationship (pattern) of the binding parameters among the 12 kidneys could be upheld despite shorter imaging times. Factor loadings of 0.800 or higher on the first factor were used as criteria satisfying this hypothesis. For factor analysis factor extraction was obtained by principal component analysis, and factor rotation was carried out by the Varimax technique (IBM SPSS Statistics, IBM Corporation Software Group Somers, NY, USA).

## 3. Results

### 3.1. Hemodynamic Effects

**Figure 3:**
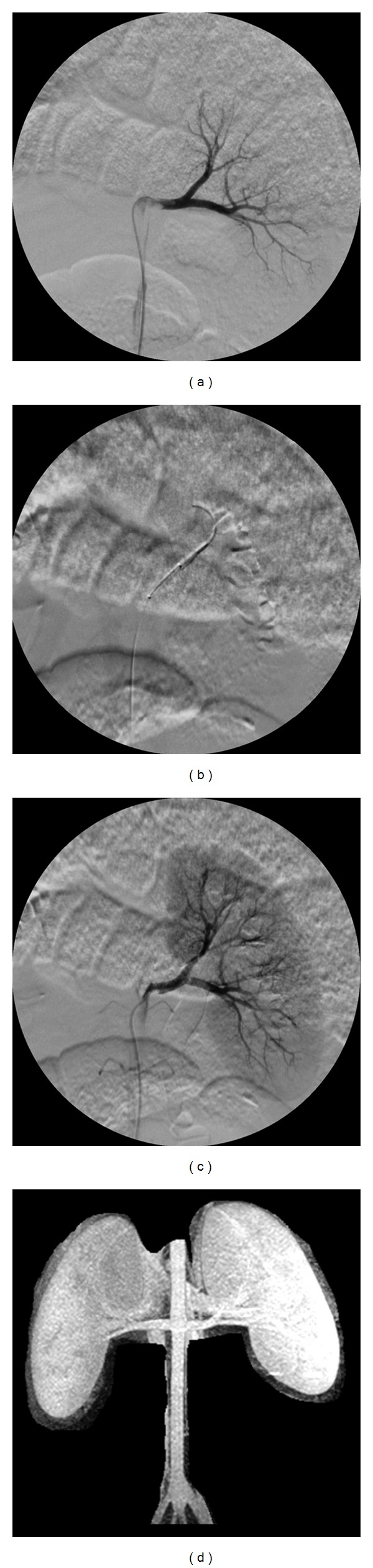
Digital angiograms obtained before, during, and after balloon occlusion of the renal artery. Baseline angiogram was recorded to check the initial status of the renal arteries (a). The balloon was inflated within the left renal artery for 60 minutes to mimic acute occlusion (b). The balloon was deflated to allow reperfusion of the left renal artery (c). Maximum intensity projection (MIP) image from renal MRA one week after IR showed bilateral patent renal arteries and symmetric gadodiamide accumulation within the kidneys (d).

Angiography confirmed successful occlusion of the left renal artery in the animals that underwent the procedure for 60 minutes as well as complete reopening on reperfusion (Figures 3(a)–3(c)). One week after intervention MRA images demonstrated patent bilateral renal arteries in both the IR and the control animals (Figure 3(d)). The [^15^O]H_2_O PET study showed no significant difference suggesting anatomical interruption of blood flow (simple *t*-test *P* > 0.05) between the control kidneys (perfusion ratio 0.865 ±  0.093) and IR kidneys (perfusion ratio 0.809 ±0.214) although there was a slight perfusion predominance of the right kidney in all animals. No significant effect of IR was observed on the levels of angiotensin II, plasma renin activity (PRA), and aldosterone (*P* > 0.05) (Table 1) or on the values of blood chemistries and urine specific gravity and PH values (*P* > 0.05) (Table 2). Therefore there was no systemic chemical or hormonal evidence to suggest the presence of renovascular hypertension or any degree of renal failure.

### 3.2. *In Vitro* Receptor Binding

**Figure 4:**
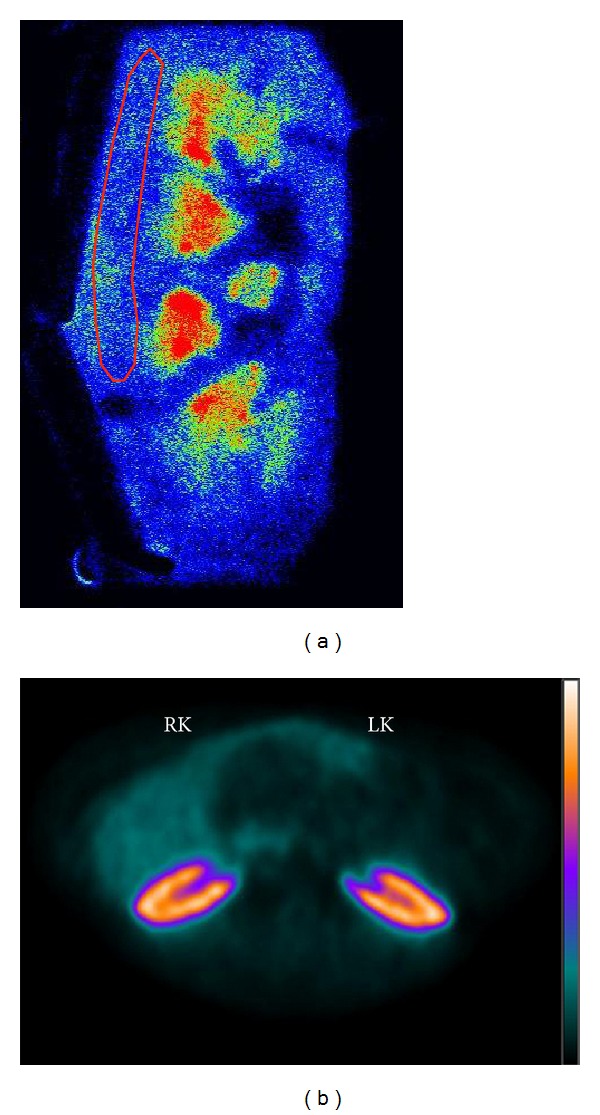
Examples of digital autoradiography (a) and a PET scan (b). Digital autoradiography shows clear distinction between cortex and medulla. Although radioligand binding in the medulla is higher than that in the cortex, a cortical ROI was used for data analysis to correlate *in vitro* binding with *in vivo* cortical binding. The PET scan represents an axial summed image between 30 and 60 minutes after injection. There was no difference between [^11^C]KR31173 binding in the left kidney after IR (LK) and the contralateral right kidney (RK).

**Figure 5:**
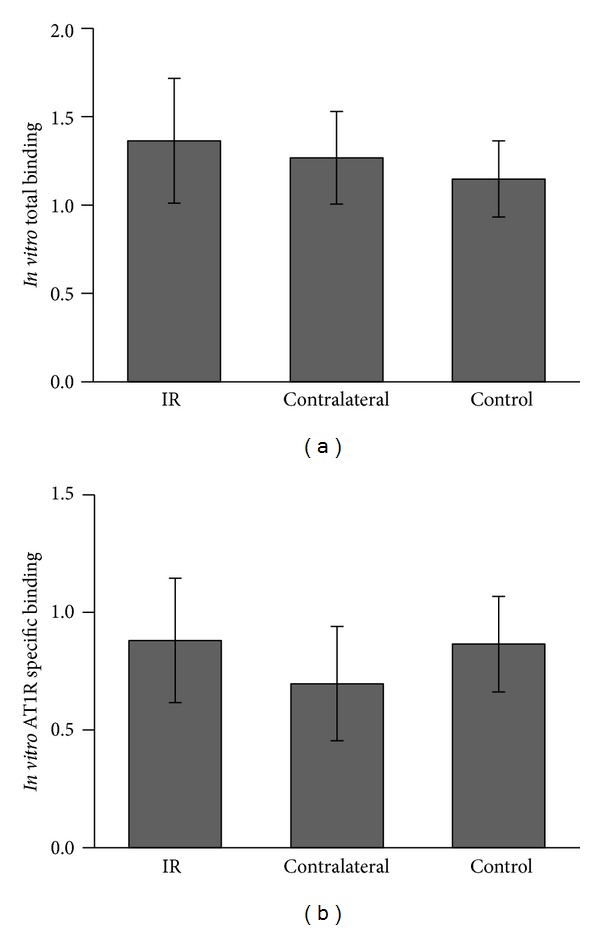
*In vitro* radioligand (a) total binding and (b) AT1R specific binding determined by digital autoradiography. The differences between IR, CL, and C kidneys are not significant (*P* > 0.05).

A typical autoradiography image of a coronal slice of the whole kidney is shown in Figure 4(a). The image shows higher binding in the medulla. Since under *in vivo* conditions radioligands predominantly depict the renal cortex [14], the region of interest (ROI) for quantitative analysis was placed over the cortex. There was no significant difference in total or specific *in vitro* binding of [^125^I]-[Sar^1^Ile^8^] detected by autoradiography between IR kidneys, kidneys contralateral to IR kidneys, and control kidneys (Figure 5). AT1R specific binding was defined as [^125^I]-[Sar^1^Ile^8^] Ang II binding after tissue incubation with the AT2R antagonist PD-123,319 and in actual fact included AT1R binding as well as non-AT1R/non-AT2R binding.

### 3.3. *In Vivo* Radioligand Binding

**Figure 6:**
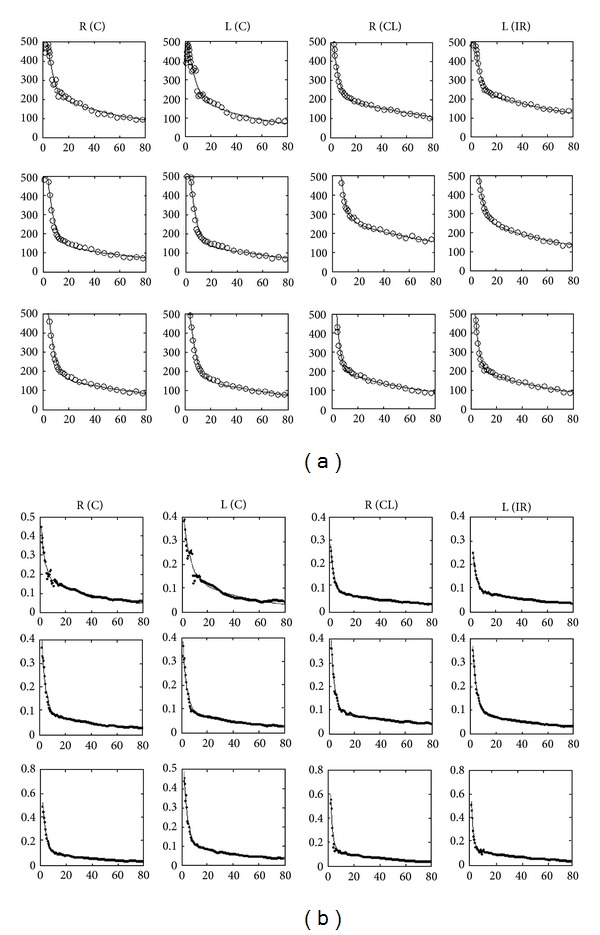
Compartmental curve fits of time-activity curves (a) and impulse response function (b). Visually both the TACs and the impulse response functions appear biexponential. In the compartmental model the fit is excellent for each kidney with both approaches.

**Figure 7:**
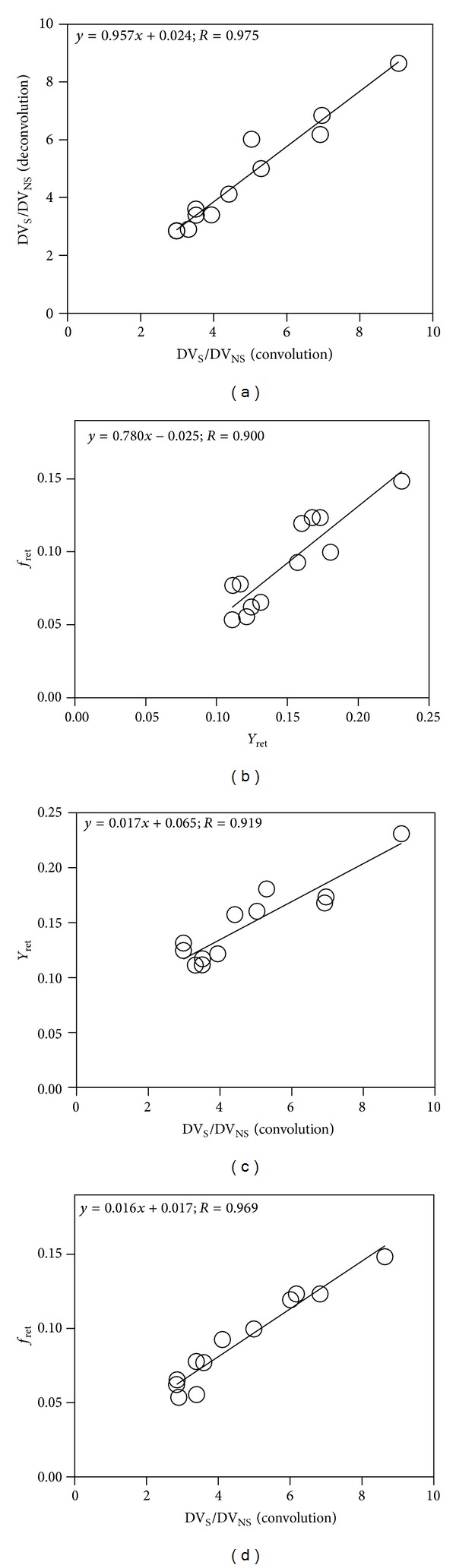
Parameter correlations. (a) Correlation of distribution volume ratios DVR obtained with the convolution and deconvolution approaches is significant and confirms robustness of the parallel model. (b) Correlation between radioligand retention values from the time-activity curve (*Y*_ret_) and from the impulse response function (*f*_ret_) shows that the noninvasive parameter (*Y*_ret_) represents a good estimation of the invasive parameter (*f*_ret_). (c) Correlation between *Y*_ret_ and DV_S_/DV_NS_ from convolution shows that radioligand retention obtained from time-activity curve noninvasively can be used for estimation of the invasive displaceable to nondisplaceable binding ratio. (d) Correlation between *f*_ret_ and DV_S_/DV_NS_ from convolution shows that radioligand retention obtained from the impulse response function can also be used for estimation of the distribution volume ratios.

Transaxial PET scan images were used for ROI placement and curve generation for quantification of *in vivo* binding (Figure 4(b)). Excellent curve fits were achieved both with the convolution approach (Figure 6(a)) and the deconvolution approach (Figure 6(b)). The impulse response function was unequivocally biexponential in shape (Figures 6(a) and 6(b)), in line with a two-tissue compartment model. The marked dissimilarity between the fast (nonspecific) and slow (specific) binding components was also consistent with a parallel model. Three-way comparisons of parameters showed no significant group effects between C, IR, and CL kidneys. The ANOVA *F* values were between 0.111 (*P* = 0.896) and 3.088 (*P* = 0.095). Also, two-way comparisons revealed no parameter differences between IR and C kidneys or between CL and C kidneys (Table 3). Correlations between the corresponding binding parameters derived by the convolution and deconvolution approaches were significant (*r* = 0.845–0.993) concurrently with the significant correlation between *Y*_ret_ and *f*_ret_ (Table 4; *r* = 0.900). The most important correlations are displayed as scatterplots with the fitted regression lines for the following parameters: DVR convolution versus DVR deconvolution (Figure 7(a)), *Y*_ret_ versus *f*_ret_ (Figure 7(b)), and DVR convolution versus *Y*_ret_ (Figure 7(c)), DVR deconvolution versus *f*_ret_ (Figure 7(d)). Each one of these correlations is significant (*r* > 0.9).

The most reliable (reference) value of DVR was calculated including data points up to 85.7 min (total imaging time 90 min). The effects of shortened imaging times were investigated by calculating the DVR using earlier time points and comparing them to the reference. The bias of DVR was less than 10% for time points 57.5 min and higher (total imaging time at least 60 min) while the bias of *Y*_ret_ was less than 10% for time points 82.5 min or higher (total imaging time at least 85 min). Principal component based factor analysis of DVR values of the 12 kidneys obtained with multiple time points showed that with two factors 99% of the total variance could be explained for DVR and 96% of the total variance could be explained for *Y*_ret_. All values of DVR calculated at time points 47.5 min or longer showed mutual correlations with loading values on the first factor of >0.9 (Table 5).

The reference value of the retention parameter *Y*_ret_ was calculated using the 85.7 min time point (total imaging time of 90 min). The effects of shortened imaging times were obtained by calculating the *Y*_ret_ at earlier time points and by analyzing the set of *Y*_ret_ values by factor analysis. The results for *Y*_ret_ were less favorable than the results for DVR. Although the correlation between the *Y*_ret_ values obtained with different end time points was high (factor loadings 0.843–0.977), an acceptable bias of less than 10% was only achieved at time points 82.5 and 87.5 min (total imaging times of 85 and 95 min). This corresponded to a minimal required imaging time of 85 minutes (Table 6).

## 4. Discussion

The goal of this project was to investigate the applicability of a tracer kinetic model that is based on three compartments (one blood and two tissue compartments connected in parallel) for quantitative analysis of the renal kinetics of [^11^C]KR31173. Previously, tissue binding of this radioligand was described by the retention parameter calculated from the ratio of the 80 min value divided by the maximal value of the impulse response function. The retention parameter was found to be increased in renal ischemia, an important *in vivo* finding that was confirmed by *in vitro* autoradiography which demonstrated increased binding of [^125^I]-[Sar^1^Ile^8^] Ang II in ischemic kidneys [2].

The parallel model applied here is different from the typical kinetic models of brain PET receptor studies. For brain PET receptor studies the established catenary tracer kinetic model consists of one extracerebral compartment represented by blood and two intracerebral compartments represented by nonspecific (nonreceptor) binding and specific (receptor) binding. In early brain receptor studies a mammillary model [15] was proposed which included four compartments: (1) an extracellular free radioligand compartment which was connected via bidirectional tracer transfer with three additional compartments, (2) plasma, (3) nonspecific binding sites, and (4) specific binding sites [4]. The four-compartmental model was not applicable for most radioligands due to an insignificant difference between the turnover rates of the free ligand and the nonspecific binding compartments. As a consequence, the two compartments were combined together changing the compartment model structure from mammillary to catenary.

An overly complex model results in over parameterization with a lack of ability to discern the individual compartments and estimate their rate constants robustly. Model simplification is achieved by combining compartments together at rapid equilibrium or with kinetics computationally inseparable relative to the data acquisition time. In brain receptor PET studies the most effective simplification was achieved by combining the compartment of free radioligand with the compartment of nonspecific binding for multiple reasons; the two compartments are located on the tissue side of the blood brain barrier, the two compartments are either at or in rapid equilibrium, or the two components have similar turnover rates.

The most important rationale for the serial connectivity model in PET studies of the brain is the blood brain barrier. The radioligand has to cross this barrier before it is distributed in the tissue and binds to the receptors. Model identification and parameter estimation are complicated by the fact that the impulse response function of the serial model does not directly reflect the individual impulse response functions. Its components are mutually affected by the process of convolution. The slow nonspecific binding turnover prolongs specific binding and vice versa. 

A more simple parallel connectivity model is therefore proposed here for the kidney. In the kidneys there is no blood tissue barrier involved, and instantaneous binding occurs at the endothelial surface of the vasculature and glomeruli with both specific and nonspecific binding sites [16]. Previously, we have demonstrated high density of the AT1R in cortical glomeruli [17]. The resulting impulse response function is a linear combination of the two individual impulse response functions. Both are weighted by their association rate constants *K*_1_ and *K*_3_. Urinary excretion of [^11^C]KR31173 is minimal [18]. Computationally the parallel model requires only one convolution step between the arterial input function with the impulse response function. Both in the time-activity curves (Figure 6(a)) and in the impulse response function (Figure 6(b)) the separation of the two binding components was visually apparent and computationally straightforward. 

Measured tissue time-activity curves are often significantly affected by the input function. The effect of the input function is removed by the process of deconvolution which results in an ideal time-activity curve called the impulse response function. The impulse response functions of the 12 kidneys are shown in Figure 6(b). The large difference in the amplitude and washout of the two components again implicates parallel connectivity. The vascular compartment that would appear as a separate peak is indiscernible from the fast nonspecific binding component, which points toward high first pass extraction of the radioligand. The ratio of *K*_1_ to *K*_3_ is approximately 4 : 1. 

In this study [^15^O] water was only used to estimate relative blood flow differences between the two kidneys of the same animal assuming that one kidney is always normally perfused. Arterial blood sampling for absolute quantification was not attempted in order to minimize the effects of blood loss on the renin angiotensin system. One could, however, consider using the peak value of the impulse response function for estimation of renal blood flow. The average total first pass uptake (*K*_1_ + *K*_3_) was 1.324 ± 0.588 mL/min/mL, which corresponds to an average renal blood flow of 1.891 mL/min/g assuming an organ density of 1 g/mL and an arterial blood hematocrit of 0.3. This average value is comparable to measurements obtained with [^64^Cu]-ETS, a radioligand recently used for quantification of renal blood flow with PET [19].

The average *k*_2_ was 0.309 ± 0.107 min^−1^ equivalent to an average turnover time of 3.5 min since the two parameters are reciprocal. This turnover time includes nonspecific binding and may also include tubular excretion. However, since only one fast component was detected (Figure 6(b)), there was no way to separate nonspecific binding from urinary excretion. Furthermore, the fast component was monoexponential without a hint of a plateau or delay time to imply tubular excretion. Thus, this model included no excretory component although such a component may become more relevant in urinary obstruction, for example, situations where transport in the tubular lumen and collective system is delayed. The ratio between *K*_1_ and *K*_3_ was 4.9.

The average value of the parameter *k*_4_ from the convolution approach was 0.013 ± 0.002 min⁡⁡^−1^ corresponding to a mean turnover time of 82 min. This is more than 20 times longer than the turnover time of the fast component, a difference that facilitates differentiation of nonspecific binding (high capacity/low affinity) from specific (low capacity/high affinity) binding. Selectivity of [^11^C]KR31173 for AT1R has previously been demonstrated [2].

Parameter estimation with the deconvolution approach includes two steps. In the first step the impulse response function is calculated by deconvolution analysis [20]. In the second step the parameters are estimated by least squares fitting of the impulse response function defined in (3). Although this two-step approach appears more complex than the convolution approach, it offers the advantage of a visual investigation of the impulse response function (Figure 6(b)). A second advantage of the deconvolution approach is that for least squares curve fitting the impulse response function is calculated analytically eliminating the need for numerical integration. The similarity between the biexponential shapes of the TACs and impulse response functions can be explained by a short bolus resulting in rapid elimination of the radioligand from the circulation. 

Another advantage of the parallel compartmental model is that two distribution volumes (DV_S_ and DV_NS_) can be calculated from their rate constants and used for calculation of the distribution volume ratio DVR. The average DVR was 4.831 ± 1.922 and on an individual basis showed a high correlation between the two tested computational algorithms (Figure 7(a)). This observation further corroborates the robustness of the parallel connectivity model.

The retention parameter *Y*_ret_ also correlated with DVR obtained by convolution while *f*_ret_ correlated with DVR obtained by deconvolution (Figures 7(c) and 7(d)). There was also a positive correlation between *Y*_ret_ and *f*_ret_ (Figure 7(b)) which is explained by a short input function. This short input function is the result of multiple factors: (a) rapid injection, (b) rapid removal of the radioligand from circulation, (c) lack of significant recirculation, and (d) lack of significant radioligand metabolism.

Factor analysis demonstrated that the same pattern between the 12 kidneys could be obtained for both *Y*_ret_ and DVR when the total measurement time was 45 min or longer. The bias of DVR was less than 10% for imaging times of 60 min or longer. However, to achieve less than 10% bias in *Y*_ret_ a total imaging time of at least 85 min was required. In other words, without compartmental modeling the total scanning time has to be at least 85 min while with compartmental modeling the total time can be shortened to 60 minutes. The factor loads also demonstrated that reproducibility of DVR was higher than reproducibility of *Y*_ret_. Therefore, compartmental modeling can help shorten the imaging time from 90 min to 60 min at acceptable parameter bias and acceptable pattern reproducibility. Shortening of imaging times and avoidance of arterial blood sampling will facilitate application of this novel receptor imaging technique in humans. Further studies are needed to investigate whether an image derived input function can replace the input function obtained by arterial blood sampling. 

Previously our group demonstrated increased AT1R binding in chronic renal artery stenosis with no reperfusion in pigs [2, 21, 22]. The data presented here show that 60 minutes of renal artery occlusion did not result in significant changes in AT1R receptor binding one week after IR. Renal artery occlusion for 60 minutes has been used by several authors [23, 24]. The lack of effect should be considered reliable since the results with *in vitro* and *in vivo* studies were comparable although these are two entirely different techniques: the first one (*in vitro* autoradiography) is independent and the second one (*in vivo* PET) is dependent on tracer delivery by circulatory transport.

## 5. Conclusion

The presented data support the applicability of the three-compartment model with two tissue compartments connected in parallel for description of the kinetics of the AT1R selective radioligand [^11^C]KR31173 in the kidneys. Computationally, this model is less complex than the serial model and provides quantitative estimates of the ligand kinetic parameters and ligand binding parameters. This model will facilitate molecular mapping of renal AT1R and address scientific hypotheses on the *in vivo* regulation in clinical settings of renal hypoperfusion, transplant nephropathy, and obstructive nephropathy.

## Figures and Tables

**Table 1:** Hormone levels at baseline and one week after ischemia followed by reperfusion (IR).

		Angiotensin II	Plasma renin activity	Aldosterone
		(ng/L)	(ng/mL/h)	(ng/dL)
Baseline	Mean	22.20	0.62	5.60
	(sd)	(6.76)	(0.44)	(3.36)
IR	Mean	18.2	0.42	2.4
	(sd)	(2.77)	(0.41)	(0.89)

P*		0.18	0.53	0.13

*Differences tested by a paired *t-*test are statistically not significant (*P* > 0.05).

**Table 2:** Blood cell counts and blood chemistries at baseline and one week after ischemia followed by reperfusion (IR)*.

		WBC (Thous/uL)	RBC (millions/uL)	HB g/dL	HCT %	ALP (U/L)	sGPT (U/L)	sGOT (U/L)	CK (U/L)	BUN mg/dL	Cre mg/dL	Ca++ mg/dL	U SG (g/L)	U PH
Baseline	Mean	14.60	6.49	10.72	34.96	82.40	29.40	26.00	646	8.80	1.18	9.64	1016	6.63
	(sd)	(3.16)	(1.37)	(2.10)	(7.33)	(9.76)	(7.77)	(10.70)	(642)	(4.87)	(0.23)	(0.59)	(13)	(0.48)
IR	Mean	12.36	6.63	11.18	34.78	48.20	52.80	77.80	1257	10.80	1.22	9.26	1006	6.80
	(sd)	(2.49)	(1.69)	(2.63)	(9.23)	(27.93)	(23.39)	(76.98)	(1332)	(2.28)	(0.19)	(0.44)	(3)	(0.27)

*P *		0.2859	0.1421	0.1333	0.1638	0.6216	0.0167	0.1702	0.3811	0.1489	0.2212	0.2026	0.1878	0.1673

*Differences tested by a paired *t*-test are statistically not significant (*P* > 0.05).

WBC: white blood cells. RBC: red blood cells. HB: hemoglobin. ALP: alkaline phosphatase. sGPT: serum glutamic pyruvic transaminase. sGOT: serum glutamic oxaloacetic transaminase. CK: serum creatine kinase. BUN: blood urea nitrogen. Cre: serum creatinine. U SG: urine specific gravity. U PH: urine PH.

**Table 3:** Compartmental rate constants and parameters of ligand binding. *K*_1_, *k*_2_, *K*_3_, and *k*_4_ represent compartmental rate constants, DV_NS_ is the distribution volume of nonspecific binding, DV_S_ is the distribution volume of specific binding, *Y*_ret_ is ligand retention calculated from tissue activity, and *f*_ret_ is ligand retention calculated from the impulse response function. Further details of computations are described in the methods.

	Convolution fit								Deconvolution fit							
Group	*K*_ 1_	*k*_ 2_	*K*_ 3_	*k*_ 4_	DV_NS_	DV_S_	DVR	*Y*_ ret_ (80 min)	*K*_ 1_	*k*_ 2_	*K*_ 3_	*k*_ 4_	DV_NS_	DV_S_	DVR	*f*_ ret_ (80 min)
Mean IR^a^	1.113	0.357	0.193	0.013	2.968	15.270	5.808	0.170	0.720	0.411	0.101	0.013	1.633	7.604	5.383	0.099
(sd IR)^a^	0.731	0.131	0.040	0.003	1.058	0.844	2.830	0.056	0.523	0.146	0.019	0.002	0.665	0.653	2.844	0.047
Mean CL^b^	1.413	0.387	0.183	0.011	3.407	16.167	5.181	0.153	0.852	0.432	0.098	0.013	1.836	7.788	4.691	0.092
(sd CL)^b^	0.985	0.137	0.036	0.003	1.233	1.481	1.807	0.037	0.604	0.136	0.019	0.003	0.747	0.877	1.661	0.036
Mean C^c^	0.985	0.246	0.212	0.013	3.959	15.902	4.168	0.136	0.591	0.319	0.130	0.017	1.849	7.267	4.255	0.087
(sd C)^c^	0.250	0.031	0.077	0.002	0.628	4.058	1.564	0.025	0.160	0.030	0.056	0.005	0.476	1.313	1.726	0.027
*t* (IR vs C)^d^	0.794	0.279	0.640	0.824	0.240	0.727	0.427	0.410	0.714	0.389	0.298	0.164	0.649	0.623	0.576	0.726
*t* (CL vs C)^e^	0.532	0.215	0.467	0.417	0.527	0.891	0.459	0.528	0.534	0.283	0.245	0.126	0.980	0.506	0.732	0.853

Mean and standard deviation values for ^a^ischemia followed by reperfusion, ^b^contralateral, and ^c^control kidneys.

^d^Simple *t*-test of the differences between kidneys with ischemia followed by reperfusion (IR) and controls. ^e^Simple *t-*test of the differences between contralateral kidneys and controls. The differences are not significant (*P* > 0.05).

**Table 4:** Correlations between parameters and distribution volumes obtained with the two fitting methods (e.g., convolution versus deconvolution). All correlations are significant (*P* < 0.005). Also the correlation between the ligand retention parameters *Y*_ret_ and *f*_ret_ is significant (*P* < 0.005).

	*K*_ 1_	*k*_ 2_	*K*_ 3_	*k*_ 4_	DV_NS_	DV_S_	DVR	*Y*_ ret_ versus *f*_ret_
Correlations	0.993	0.969	0.971	0.845	0.913	0.925	0.975	0.900
*P* (1 tailed) <	0.001	0.001	0.001	0.003	0.001	0.001	0.001	0.001

**Table 5:** Effect of imaging time on the distribution volume ratio DVR. The bias represented by the average % difference (for the 12 kidneys) between the actual DVR and reference DVR is acceptable (less than 10%) at time points 57.5 min or later. Loadings on factor 1 are acceptable (higher than 0.800) at time points 47.5 min or later.

Time point (min)	42.5	47.5	52.5	57.5	62.5	72.5	72.5	77.5	82.5	87.5
Bias (%)	66	25	16	7	5	4	1	1	1	0
Factor 1 loading	0.274	0.971	0.988	0.997	0.996	0.993	0.994	0.996	0.995	0.992
Factor 2 loading	0.961	0.125	0.018	−0.064	0.007	−0.071	−0.077	−0.050	−0.065	−0.085

**Table 6:** Effect of imaging time on the distribution volume ratio *Y*_ret_. The bias represented by the average % difference (for the 12 kidneys) between the actual *Y*_ret_ and reference *Y*_ret_ is acceptable (less than 10%) at time points 82.5 min or later. Loadings on factor 1 are acceptable (higher than 0.800) at all time points.

Time point (min)	42.5	47.5	52.5	57.5	62.5	72.5	72.5	77.5	82.5	87.5
Bias (%)	53	46	40	32	25	22	18	12	9	0
Factor 1	0.877	0.898	0.977	0.973	0.967	0.986	0.963	0.975	0.843	0.8573
Factor 2	0.453	0.423	−0.105	−0.068	−0.104	−0.116	−0.205	−0.102	−0.499	0.376

## References

[1] Mathews WB, Yoo S-E, Lee S-H (2004). A novel radioligand for imaging the AT1 angiotensin receptor with PET. *Nuclear Medicine and Biology*.

[2] Xia J, Seckin E, Xiang Y (2008). Positron-emission tomography imaging of the angiotensin II subtype 1 receptor in swine renal artery stenosis. *Hypertension*.

[3] Wang ZJ, Szabo Z, Lei P, Varga J, Liu KJR (2005). A factor-image framework to quantification of brain receptor dynamic PET studies. *IEEE Transactions on Signal Processing*.

[4] Frost JJ, Douglass KH, Mayberg HS (1989). Multicompartmental analysis of [^11^C]-carfentanil binding to opiate receptors in humans measured by positron emission tomography. *Journal of Cerebral Blood Flow and Metabolism*.

[5] Derweesh IH, Novick AC (2005). Mechanisms of renal ischaemic injury and their clinical impact. *British Journal of Urology*.

[6] Loverre A, Capobianco C, Stallone G (2007). Ischemia-reperfusion injury-induced abnormal dendritic cell traffic in the transplanted kidney with delayed graft function. *Kidney International*.

[7] Rosivall L (2009). Intrarenal renin-angiotensin system. *Molecular and Cellular Endocrinology*.

[8] Higuchi T, Fukushima K, Xia J (2010). Radionuclide imaging of angiotensin II type 1 receptor upregulation after myocardial ischemia-reperfusion injury. *Journal of Nuclear Medicine*.

[9] Hilton J, Yokoi F, Dannals RF, Ravert HT, Szabo Z, Wong DF (2000). Column-switching HPLC for the analysis of plasma in PET imaging studies. *Nuclear Medicine and Biology*.

[10] Marquardt D (1963). An algorithm for least-squares estimation of nonlinear parameters. *Journal of the Society for Industrial and Applied Mathematics*.

[11] Levenberg K (1944). A method for the solution of certain problems in least squares. *Quarterly of Applied Mathematics*.

[12] Sutton DG, Kempi V (1992). Constrained least-squares restoration and renogram deconvolution: A comparison by simulation. *Physics in Medicine and Biology*.

[13] Bagby SP, Lebard LS, Luo Z (2002). ANG II AT1 and AT2 receptors in developing kidney of normal microswine. *American Journal of Physiology*.

[14] Gibson RE, Beauchamp HT, Fioravanti C, Brenner N, Burns HD (1994). Receptor binding radiotracers for the angiotensin II receptor: Radioiodinated [Sar1, Ile8]angiotensin II. *Nuclear Medicine and Biology*.

[15] De Biasi J (1989). Four open mammillary and catenary compartment models for pharmacokinetics studies. *Journal of Biomedical Engineering*.

[16] Bagby SP, LeBard LS, Luo Z, Speth RC, Ogden BE, Corless CL (2002). Angiotensin II type 1 and 2 receptors in conduit arteries of normal developing microswine. *Arteriosclerosis, Thrombosis, and Vascular Biology*.

[17] Szabo Z, Speth RC, Brown PR (2001). Use of positron emission tomography to study AT1 receptor regulation in vivo. *Journal of the American Society of Nephrology*.

[18] Zober TG, Mathews WB, Seckin E (2006). PET imaging of the AT1 receptor with [^11^C]KR31173. *Nuclear Medicine and Biology*.

[19] Green MA, Mathias CJ, Willis LR (2007). Assessment of Cu-ETS as a PET radiopharmaceutical for evaluation of regional renal perfusion. *Nuclear Medicine and Biology*.

[20] Szabo Z, Nyitrai L, Sondhaus C (1987). Effects of statistical noise and digital filtering on the parameters calculated from the impulse response function. *European Journal of Nuclear Medicine*.

[21] Kim SE, Scheffel U, Szabo Z (1996). In vivo labeling of angiotensin II receptors with a carbon-11-labeled selective nonpeptide antagonist. *Journal of Nuclear Medicine*.

[22] Zober TG, Fabucci ME, Zheng W (2008). Chronic ACE inhibitor treatment increases angiotensin type 1 receptor binding in vivo in the dog kidney. *European Journal of Nuclear Medicine and Molecular Imaging*.

[23] Allred AJ, Chappell MC, Ferrario CM, Diz DI (2000). Differential actions of renal ischemic injury on the intrarenal angiotensin system. *American Journal of Physiology*.

[24] Kontogiannis J, Burns KD (1998). Role of AT1 angiotensin II receptors in renal ischemic injury. *American Journal of Physiology - Renal Physiology*.

